# Comparison between selective caudal fixed screw construct and all variable screw construct in anterior cervical discectomy and fusion

**DOI:** 10.1038/s41598-021-90121-w

**Published:** 2021-05-19

**Authors:** Jae Jun Yang, Sehan Park, Seongyun Park

**Affiliations:** Department of Orthopedic Surgery, Dongguk University Ilsan Hospital, 14 Siksadong, Ilsandonggu, Goyangsi, Gyeonggido 411-773 Republic of Korea

**Keywords:** Orthopaedics, Risk factors

## Abstract

This retrospective comparative study aimed to compare the efficacy of selective caudal fixed screw constructs with all variable screw constructs in anterior cervical discectomy and fusion (ACDF). Thirty-five patients who underwent surgery using selective caudal fixed screw construct (SF group) were compared with 44 patients who underwent surgery using all variable constructs (AV group). The fusion rate, subsidence, adjacent level ossification development (ALOD), adjacent segmental disease (ASD), and plate-adjacent disc space distance were assessed. The one-year fusion rates assessed by computed tomography bone bridging and interspinous motion as well as the significant subsidence rate did not differ significantly between the AV and SF groups. The ALOD and ASD rates and plate-adjacent disc space distances did not significantly differ between the two groups at both the cranial and caudal adjacent levels. The number of operated levels was significantly associated with pseudarthrosis in the logistic regression analysis. The stability provided by the locking mechanism of the fixed screw did not lead to an increased fusion rate at the caudal level. Therefore, the screw type should be selected based on individual patient’s anatomy and surgeon’s experience without concern for increased complications caused by screw type.

## Introduction

Anterior cervical plating has been widely applied in anterior cervical discectomy and fusion (ACDF) to enhance fusion rate, improve cervical alignment, and prevent graft subsidence^1–5^. However, the anterior cervical plate is not without implant-related complications, including screw migration and fracture^6–8^. Furthermore, a plate placed proximal to the adjacent disc space reportedly increases the incidence of adjacent level ossification development (ALOD), which can adversely affect the range of motion and degeneration of unoperated levels^9–11^. Therefore, plating techniques that can minimize the chance of complications while taking advantages are needed^10,12,13^.

Dynamic plating has been commonly used because it can avoid distraction force at the graft–bone interface and stress shielding^14^. The fixed and variable screws are two types of screws used for dynamic plating. Because the insertion angle is more freely adjustable with variable types, endplate injury can be avoided with a variable screw even when the screw insertion point is near the operated disc space. Therefore, a variable screw is advantageous when keeping the plate-adjacent disc space distance to > 5 mm, which is needed to avoid ALOD^9–11^. Fixed screws provide additional stability due to the stable grabbing at the screw-plate interface. Whereas fixed screws allow rigid fixation, variable screws allow toggling or rotational movement, which demonstrates the advantages of dynamic plating^14^.

Diverse anterior cervical plating constructs such as fixed construct, hybrid construct, and unconstrained constructs using different types of screws are being utilized^2,13^. Previous reports have demonstrated that favorable clinical outcomes with a high fusion rate can be achieved using diverse types of screw constructs^2,13^. Park et al. reported a fusion rate of 100% by using fixed screws only at the cranial vertebra and variable screws at the middle and caudal vertebrae^15^. A fusion rate of 83% has also been reported using all variable screw constructs^6^. However, most studies did not directly compare the results of different screw constructs. Although it has been reported that the fusion rate is not affected by the screw type, there is little evidence regarding the rate of ALOD, ASD, or subsidence according to screw type^16^. Therefore, whether using different types of screw constructs could lead to different outcomes needs further evaluation.

Previous reports have demonstrated that ALOD more commonly occurs at the proximal adjacent level compared to the distal adjacent level^9,10^. Furthermore, pseudarthrosis or implant failure most commonly occur at the caudal-most level^6,17,18^. Therefore, we attempted a hybrid construct using fixed screws at the caudal-most instrumented level and variable screws at the cranial and middle levels to prevent ALOD at the proximal adjacent level while minimizing the pseudarthrosis rate at the caudal level. This selective caudal fixed screw construct would maintain a cranial plate-disc space distance of > 5 mm to prevent ALOD and provide further stability at the caudal-most level by using the locking screw mechanism of fixed screw. We hypothesized that a selective caudal fixed screw construct would result in a higher fusion rate and a similar rate of ALOD or adjacent segment degeneration (ASD) as the all variable constructs.

## Materials and methods

### Statement

All procedures were performed in compliance with the standards of our department. The study protocol was approved by the Institutional Review Board of Dongguk University Ilsan Hospital (01-019). The requirement for informed consent was waived due to the retrospective nature of the study. The methods were performed in accordance with relevant guidelines and regulations. This study was designed and reported in accordance with the Strengthening the Reporting of Observational Studies in Epidemiology (STROBE) statement for cohort studies, which provides guidance for strengthening observational studies^19^.

### Patient characteristics and study design

We retrospectively reviewed 101 patients who underwent ACDF with plate augmentation between September 2012 and March 2018 who met the inclusion/exclusion criteria. Inclusion criteria were: (1) patients who had surgery due to degenerative cervical myelopathy/radiculopathy at levels between C2 and C7; (2) patients who underwent surgery using allograft as an interbody spacer; (3) those with a number of operations between one and three; and (4) those who were followed-up for at least 2 years postoperatively. Exclusion criteria were: (1) patients who underwent surgery due to tumor, infection, or trauma; (2) and those who had previous cervical operation. All operations were performed by a single surgeon (JJY).

**Figure 1:**
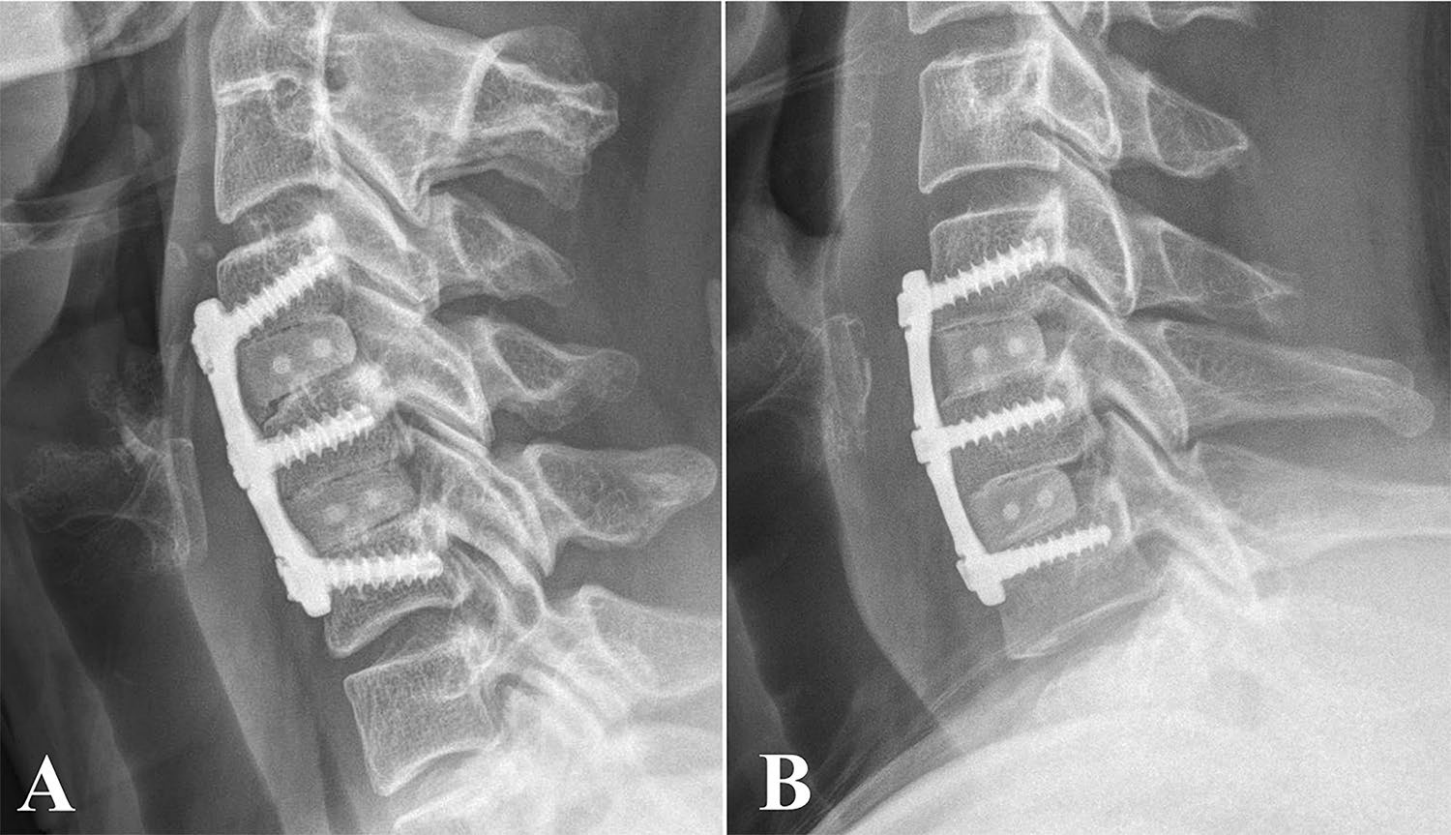
All variable screw construct and selective caudal fixed screw construct. (**A**) All variable screw construct. A short plate was selected to maintain the plate-adjacent disc space distance at > 5 mm. The screw was directed obliquely to insert the longest screw possible. (**B**) Selective caudal fixed screw construct. Fixed screws were inserted at the caudal level to increase stability. Variable screws were inserted at the cranial level and middle level. Fixed screw insertion at the caudal level allowed less caudal angulation.

Patients who underwent surgery using all the variable screw non-constrained constructs were categorized as the all variable group (AV group). Patients who underwent surgery using the hybrid construct with fixed screws at the lowermost instrumented vertebra and variable screws at rest of the levels were categorized as the selective fixed group (SF group) (Fig. 1). In our institute, all variable screw constructs were used for patients who underwent surgery before September 2017. A selective screw construct was used between October 2017 and March 2018. We originally used all variable screw constructs and changed the strategy into a selective fixed screw construct in an attempt to decrease pseudarthrosis and subsidence. The primary endpoints of the study were fusion and subsidence. ALOD, ASD, and patient-reported outcome measures, such as neck and arm pain visual analogue scale (VAS) and neck disability index (NDI) scores were defined as secondary endpoints.

### Data collection

Clinical characteristics and radiological data of patients were collected from medical chart reviews. The neck and arm pain VAS and NDI scores were recorded preoperatively, 1-year postoperatively, and at the final follow-up.

**Figure 2:**
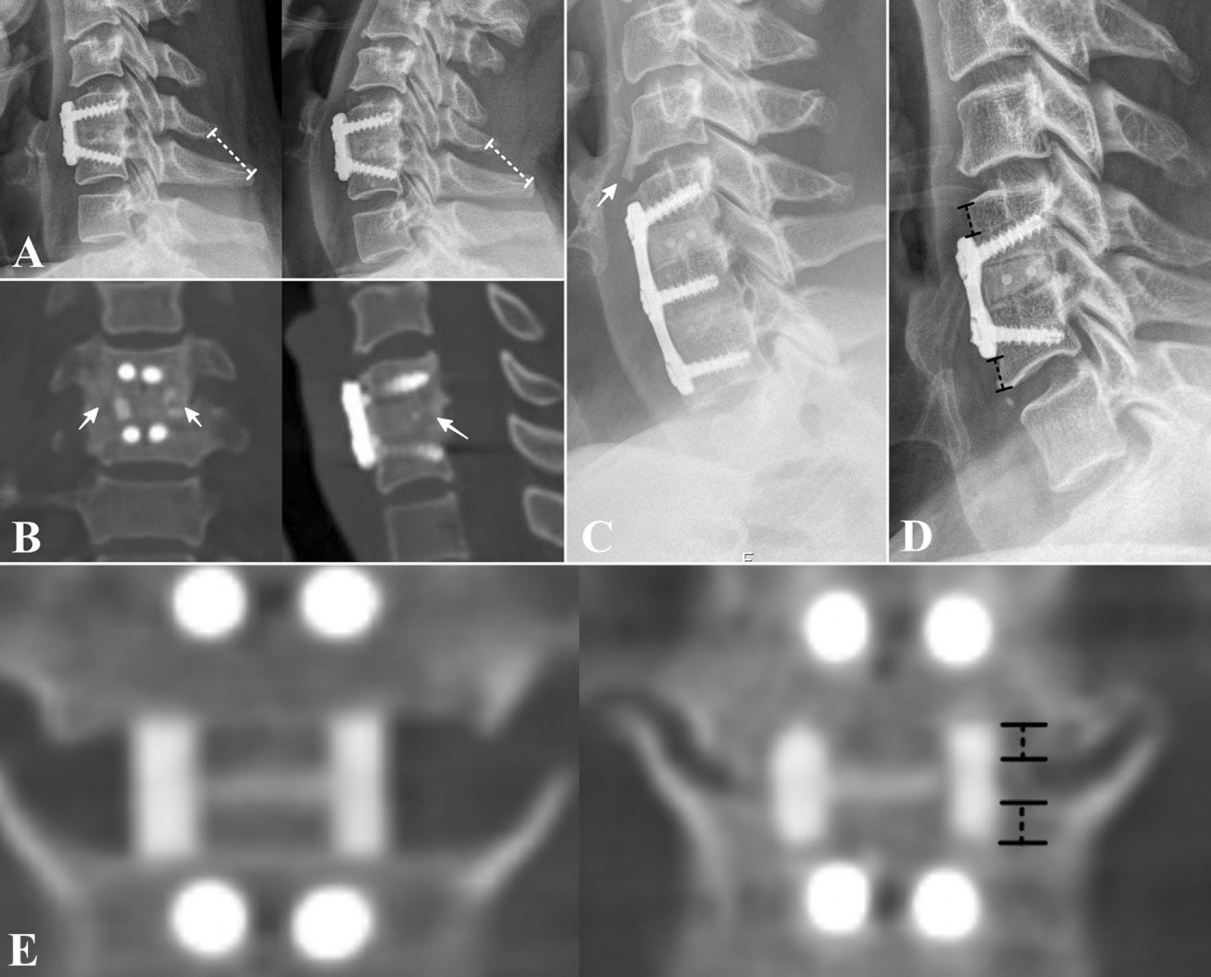
Radiographic measurements (**A**) Fusion by interspinous motion. Interspinous motion < 2 mm on 150% magnified flexion/extension lateral radiograph was considered as fusion. (**B**) Fusion was achieved by bone bridging. Bone bridging formation demonstrated both on coronal and sagittal reconstructed CT images were assessed as fusion. (**C**) Ossification of the anterior longitudinal ligament crossing the adjacent disc space demonstrated on lateral cervical radiograph was considered as an adjacent level ossification development. (**D**) Plate-adjacent disc space distance. The distance between the tips of the plate to the cephalad and caudal adjacent disc spaces on the immediate postoperative lateral radiograph of the cervical spine is shown. (**E**) Assessment of subsidence. Amount of subsidence was measured by comparing the distance between endplate of vertebral body and edge of the allograft (dotted lines) measured at CT taken 2-days and 1 year postoperatively. Subsidence of > 2 mm demonstrated in at least one of upper or lower endplate-allograft interface was defined as significant subsidence.

Radiological measurements were performed twice by a spine fellowship-trained surgeon who was not informed of the study intention previously. Cervical lordosis was measured by the angle between the lines passing through the lower margin of C2 and C6 or C7 vertebrae^20^. Fusion was assessed at 12 months postoperatively by two methods using dynamic lateral radiographs and computed tomography (CT) images using the following criteria: (1) interspinous motion (ISM) < 2 mm on a 150% magnified flexion/extension lateral radiograph (Fig. 2A^21^; and (2) bone bridging formation on sagittal and coronal reconstructed CT images (Fig. 2B)^22^. The amount of subsidence was measured by comparing the distance between the endplate of the vertebral body and the edge of the allograft measured on CT taken 2 days and 1 year postoperatively. Subsidence of > 2 mm demonstrated in at least one of the upper or lower endplate-allograft interface was defined as significant subsidence (Fig. 2E). Adjacent segments with disc height changes or osteophyte formation at the 1-year follow-up CT were identified as ASD^23^. ALOD was assessed when anterior longitudinal ligament ossification crossing the adjacent disc space was identified (Fig. 2C)^9^. The ASD and ALOD were assessed both at proximal and distal adjacent levels. The plate-adjacent disc space distance was measured as the distance between the tips of the plate to the cephalad and caudal adjacent disc spaces on the immediate postoperative lateral radiograph of the cervical spine (Fig. 2D^9,10^.

### Surgical technique

The standard Smith-Robinson approach was used to expose the indicated levels. After complete discectomy, the cartilage material was removed using a ring curette. Care was taken to achieve complete box-shaped endplate preparation. A corticocancellous allograft (Cornerstone ASR, Medtronic, Minneapolis, MN, USA) with an appropriate size was inserted into the disc space. Local autogenous bone grafts were inserted into the remaining empty disc space^24^.

For the AV group, cranial and caudal screws were inserted at the anterior endplate corners and angled away from the endplate to use the shortest cervical plate (Atlantis, Medtronic, Minneapolis, MN, USA) and longest screw to keep the cranial and caudal ends of the plate as far away from the adjacent discs as possible. All screws were inserted using the variable angle type. In the SF group, cranial screws were inserted using the same technique as described for the AV group. The fixed screws were inserted at the caudal level and variable screws were used at the rest of the levels. The insertion angle of the fixed-angle screw was 12° caudal. Screw length was determined based on preoperative CT measurements. The patients wore a neck collar for 6–12 weeks.

### Statistical analysis

Categorical variables were analyzed using the chi-square test, whereas continuous variables were analyzed using the Student’s t-test. A logistic regression analysis was performed to identify factors associated with pseudarthrosis. Further subgroup analysis comparing single-level operation and multi-level operation by chi-square test was also performed. Intraobserver agreements were assessed using the intraclass correlation coefficient (ICC) and Kappa coefficient. All data management and analyses were performed using SPSS version 21.0 software (SPSS, Inc., Chicago, IL, USA). P-values < 0.05 were considered significant.

## Results

Of the 101 patients reviewed, 79 met the inclusion criteria, and were included in the study. Forty-four patients were included in the AV group (mean age 56.1 ± 12.4 years; 26 men [59.1%]) and 35 patients were included in the SF group (mean age 56.1 ± 12.2 years; 21 men [60.0%]). The AV group involved more radiculopathy patients than the SF group (p = 0.02). In contrast, there were no baseline differences between the two groups. Number of levels operated was 1.6 ± 0.7 levels for the AV group and 1.7 ± 0.7 levels for the SF group (p = 0.75) (Table 1). One patient (2.3%) in the AV group underwent reoperation due to surgical site infection. One patient (2.9%) in the SF group underwent reoperation due to adjacent segment degeneration. All patients who were included in the study went through radiographic and CT evaluation.

**Table 1:** Patient characteristics.

	AV group	SF group	P value
Age	56.1 ± 12.4	56.1 ± 12.2	0.99
**Sex**			1.00
Male	26 (59.1%)	21 (60.0%)	
Female	18 (40.9%)	14 (40.0%)	
**Diagnosis**			0.02*
Radiculopathy	35 (79.5%)	18 (51.4%)	
Myelopathy	9 (20.5%)	17 (48.6%)	
Smoking status	10 (22.3%)	11 (31.4%)	0.45
BMI (kg/m^2^)	26.1 ± 3.9	24.7 ± 5.4	0.17
BMD (g/cm^2^)	1.0 ± 0.2	0.9 ± 0.2	0.23
Follow-up period (m)	49.8 ± 14.9	47.6 ± 30.6	0.68
Number of levels	1.6 ± 0.7	1.7 ± 0.7	0.75
**Complications**			
Dural tear	0 (0.0%)	0 (0.0%)	n/a
Hematoma	0 (0.0%)	0 (0.0%)	n/a
Infection	1 (2.3%)	0 (0.0%)	1.00
Readmission	0 (0.0%)	1 (2.9%)	0.44
Reoperation	1 (2.3%)	1 (2.9%)	1.00
Neurologic deficit	0 (0.0%)	0 (0.0%)	n/a

Age, BMI, follow-up period, number of levels were analyzed using a student’s t-test.

Sex, diagnosis, smoking status, complications were analyzed using a chi-square test.

*AV* all variable, *SF* selective caudal, *BMI* body mass index, *m* months, *n/a* not available.

*P < 0.05.

The kappa coefficient for intraobserver reliability was 0.828 for the assessment of fusion and 0.768 for the assessment of subsidence. The ICC for intraobserver reliability was 0.833 for the measurement of cervical lordosis and 0.817 for the measurement of plate-adjacent disc space distance.

### Radiographic and clinical results

**Figure 3:**
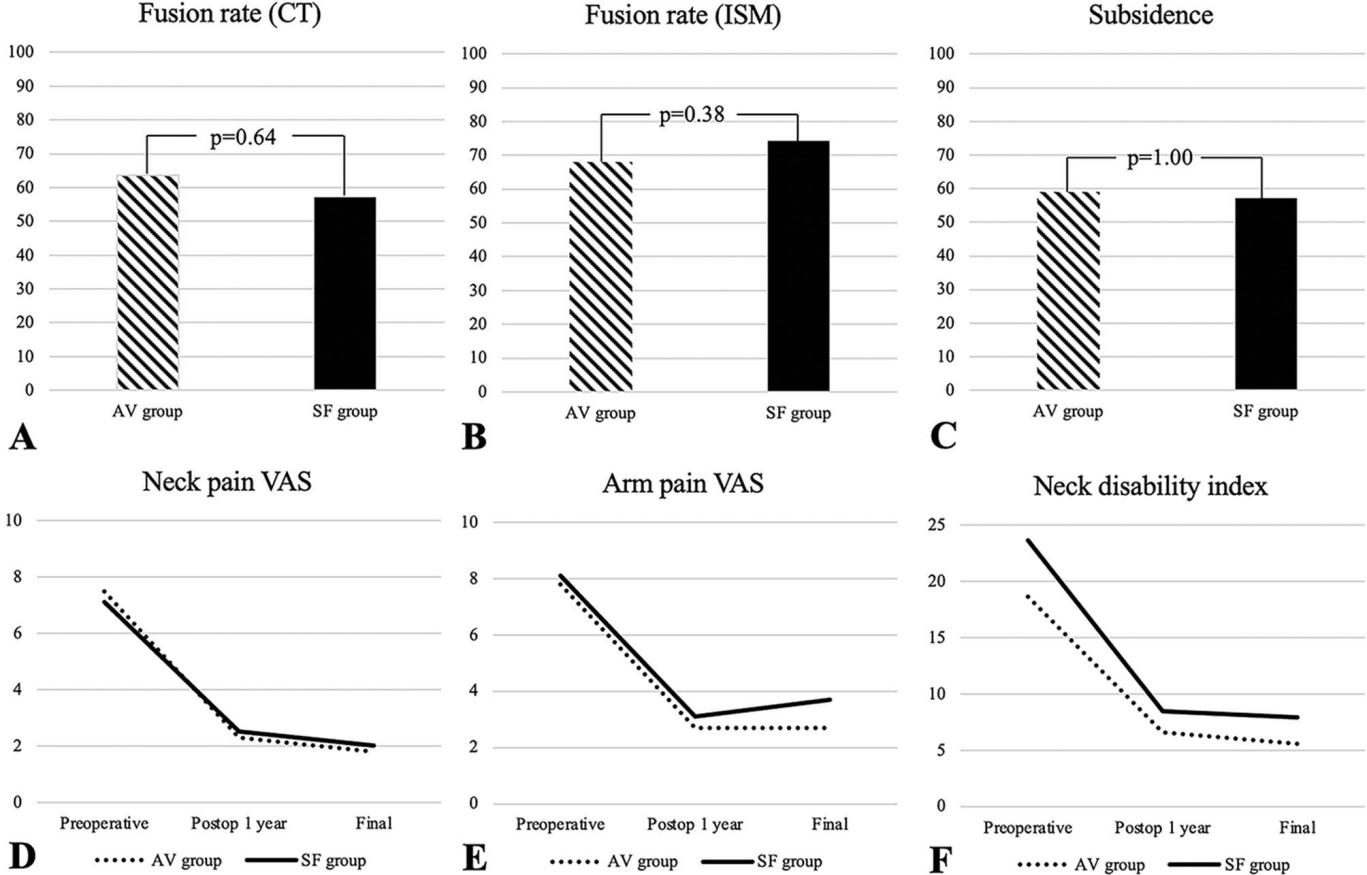
Radiographic results and patient reported outcome measures. (**A**) Fusion rates assessed by bone bridging evaluated on CT. (**B**) Fusion rates assessed by interspinous motion. (**C**) Subsidence rate (**D**). Neck pain visual analogue scale. (**E**) Arm pain visual analogue scale. (**F**) Neck disability index.

One-year fusion rates assessed by CT bone bridging (28 [63.6%] vs. 20 [57.1%], p = 0.64) and ISM (30 [68.1%] vs. 26 [74.3%], p = 0.38) did not differ significantly between the AV and SF groups (Fig. 3A,B). Furthermore, the significant subsidence rate did not differ significantly between the two groups (22 [50.0%] vs. 20 [57.1%], p = 1.00) (Fig. 3C). Subgroup analysis of fusion and subsidence rates according to the number of operated levels also did not demonstrate a significant difference between the AV and SF groups. There was no significant difference in cervical lordosis between the AV and SF groups at each follow-up period (Table 2).

**Table 2:** Radiographic results.

	AV group	SF group	P value
**Fusion CT**	28 (63.6%)	20 (57.1%)	0.64
1-level	16 (80.0%)	12 (75.0%)	1.00
2-levels	10 (50.0%)	6 (42.8%)	0.74
3-levels	2 (50.0%)	2 (40.0%)	1.00
**Fusion ISM**	30 (68.1%)	26 (74.3%)	0.38
1-level	13 (65.0%)	10 (62.5%)	1.00
2-levels	14 (70.0%)	13 (92.8%)	0.20
3-levels	3 (75.0%)	3 (60.0%)	0.64
**Subsidence**	26 (59.1%)	20 (57.1%)	1.00
1-level	12 (60.0%)	7 (43.8%)	0.50
2-levels	12 (60.0%)	9 (64.3%)	1.00
3-levels	2 (50.0%)	4 (80.0%)	0.52
**C2–C7 lordosis**			
Preoperative	12.6 ± 10.1	14.9 ± 10.5	0.34
Postoperative	17.5 ± 9.8	20.2 ± 8.6	0.20
Final follow-up	16.0 ± 9.8	17.9 ± 7.9	0.36
**Pseudarthrosis location**			0.93
Single level operation	4 (9.1%)	4 (11.4%)	
Multi-level operation			
Lowermost	11 (25.0%)	10 (28.6%)	
Other level	2 (4.5%)	3 (8.6%)	
Multiple locations	1 (2.3%)	2 (5.7%)	
**Subsidence location**			0.29
Single level operation	13 (29.5%)	7 (20.0%)	
Multi-level operation			
Lowermost	13 (29.5%)	11 (31.4%)	
Other level	9 (20.5%)	6 (17.1%)	
Multiple locations	9 (20.5%)	4 (11.4%)	

Fusion, subsidence, location of pseudarthrosis, and location of subsidence were analyzed using a chi-square test.

C2–C7 lordosis was analyzed using a student’s t-test.

*AV* all variable, *SF* selective fixed, *ISM* interspinous motion.

*P < 0.05.

Pseudarthrosis most commonly occurred in the lowermost level in the AV (11/44, 25.0%) and SF groups (10/35, 28.6%). Subsidence also most commonly occurred in the lowermost level in the AV (13/44, 29.5%) and SF groups (11/35, 31.4%). The distribution of the location of pseudarthrosis (p = 0.93) or subsidence (p = 0.29) did not significantly differ between the two groups (Table 2).

At the proximal adjacent level, 3 (6.8%) ALODs and 2 (4.5%) ASDs were detected in the AV group, whereas 3 (8.6%) ALODs and 2 (5.7%) ASDs were identified in the SF group. The rates of ALOD (p = 1.00) and ASD (p = 1.00) did not significantly differ between the two groups. Plate-adjacent disc space distance did not significantly differ between the AV and SF groups (5.5 ± 1.8. vs. 5.3 ± 1.8 mm, p = 0.60). Furthermore, the rate of plate-adjacent disc distance < 5 mm did not significantly differ between the two groups (14 [31.8%] vs. 12 [34.3%], p = 1.00) (Table 3).

**Table 3:** Radiographic results regarding ALOD and ASD.

	AV group	SF group	P value
**Proximal adjacent level**			
ALOD	3 (6.8%)	3 (8.6%)	1.00
ASD	2 (4.5%)	2 (5.7%)	1.00
Plate-adjacent disc distance (mm)	5.5 ± 1.8	5.3 ± 1.8	0.60
Plate-adjacent disc distance < 5 mm	14 (31.8%)	12 (34.3%)	1.00
**Distal adjacent level**			
ALOD	3 (6.8%)	2 (5.7%)	1.00
ASD	0 (0.0%)	0 (0.0%)	n/a
Plate-adjacent disc distance	7.4 ± 2.3	6.8 ± 2.4	0.24
Plate-adjacent disc distance < 5 mm	7 (15.9%)	10 (28.5%)	0.27

ALOD, ASD, and plate-adjacent disc distance < 5 mm were analyzed using a chi-square test;

Plate-adjacent disc distance was analyzed using a student’s t-test.

*AV* all variable, *SF* selective fixed, *ALOD* adjacent level ossification development, *ASD* adjacent segmental disease, *n/a* not available.

In the distal adjacent level, 3 (6.8%) ALODs were noted in the AV group, and 2 (5.7%) ALODs in the SF group (p = 1.00). There were no cases of ASD at the distal adjacent level in both groups. Plate-adjacent disc space distance did not significantly differ between the AV and SF groups (7.4 ± 2.3 mm vs 6.8 ± 2.4 mm, p = 0.24). The rate of plate-adjacent disc distance < 5 nm also did not differ significantly between the two groups (7 [15.9%] vs. 10 [28.5%], p = 0.27) (Table 3).

The neck and arm pain VAS and NDI scores significantly improved postoperatively in both groups. Neck and arm pain VAS and NDI scores at the 1-year postoperative follow-up (neck pain VAS, 2.3 ± 1.4 vs 2.5 ± 1.5, p = 0.46; arm pain VAS, 2.7 ± 1.6 vs 3.1 ± 1.7; p = 0.33; NDI, 6.6 ± 4.6 vs 8.5 ± 5.7; p = 0.12) and final follow-up (neck pain VAS, 1.8 ± 1.2 vs 2.0 ± 1.7, p = 0.59; arm pain VAS, 2.7 ± 1.1 vs 3.7 ± 3.6, p = 0.15; NDI, 5.6 ± 4.5 vs 7.9 ± 5.9, p = 0.08) did not significantly differ between the AV and SF groups (Table 4) (Fig. 3D–F).

**Table 4:** Patient reported outcome measure results.

		AV group	SF group	P value^†^
Neck pain VAS	Preoperative	7.5 ± 1.4	7.1 ± 2.7	0.33
	Postop 1 year	2.3 ± 1.4	2.5 ± 1.5	0.46
	Final	1.8 ± 1.2	2.0 ± 1.7	0.59
	P value^‡^ (Pre—1 year)	< 0.01	< 0.01	
	P value^‡^ (Pre—final)	< 0.01	< 0.01	
Arm pain VAS	Preoperative	7.8 ± 1.6	8.1 ± 1.5	0.62
	Postop 1-year	2.7 ± 1.6	3.1 ± 1.7	0.33
	Final	2.7 ± 1.1	3.7 ± 3.6	0.15
	P value^‡^ (Pre—1 year)	< 0.01	< 0.01	
	P value^‡^ (Pre—final)	< 0.01	< 0.01	
NDI	Preoperative	18.6 ± 4.8	23.6 ± 6.4	< 0.01*
	Postop 1-year	6.6 ± 4.6	8.5 ± 5.7	0.12
	Final	5.6 ± 4.5	7.9 ± 5.9	0.08
	P value^‡^ (Pre—1 year)	< 0.01	< 0.01	
	P value^‡^ (Pre—final)	< 0.01	< 0.01	

*AV* all variable, *SF* selective fixed, *VAS* visual analogue scale, *NDI* neck disability index, *pre* preoperative.

^†^Comparison between two groups were performed by student’s t-test.

^‡^Comparison between preoperative and postoperative values were performed by paired t-test.

*P < 0.05.

### Factors associated with pseudarthrosis

A logistic regression analysis demonstrated that the number of levels operated was significantly associated with the occurrence of pseudarthrosis assessed on CT (p = 0.01) (Table 5). A subgroup comparison between single-level operation and multi-level operation (2 or 3 levels) demonstrated that multi-level operation was associated with an increased risk of pseudarthrosis (single level, 28/36 [77.8%]; multi-level, 20/43 [46.5%]; p < 0.01).

**Table 5:** Logistic regression analysis demonstrating factors associated with nonunion.

Univariate analysis	Odds ratio	Confidence interval	P value
Age	0.99	0.951–1.025	0.50
Number of levels operated	2.52	1.224–5.175	0.01*
Smoking status	0.82	0.296–2.246	0.69
BMI	1.10	0.977–1.240	0.11
BMD	0.95	0.774–1.175	0.66
Screw construct type	0.76	0.307–1.890	0.56
Subsidence	0.52	0.202–1.328	0.17
Preoperative lordosis	0.99	0.946–1.034	0.63

*BMI* body mass index, *BMD* bone mineral density;

*P < 0.05.

## Discussion

Many previous studies have been performed to determine the optimal plating method for ACDF^2,10,12,13,16^. One issue regarding the safe plating method is the decreasing incidence of ALOD. Lee et al. reported that the plate-disc space distance should be > 5 mm to decrease ALOD incidence^10^. This technique involves inserting cranial and caudal screws from the corners immediately adjacent to their respective operative-level end plate and placing the shortest plate that fits this screw placement^10,11^. This technique, by limiting anterior longitudinal ligament dissection, is reported to decrease ALOD incidence with no additional complications^10,11^.

Other points to consider in anterior cervical plating are decreasing the amount of subsidence and the rate of pseudarthrosis. Park et al. reported that a short plate with an oblique screw trajectory construct is effective for decreasing the incidence of ALOD and subsidence^12^. A screw length greater than 75% of the antero-posterior vertebral body diameter is recommended to decrease the pseudarthrosis rate^13^.

**Figure 4:**
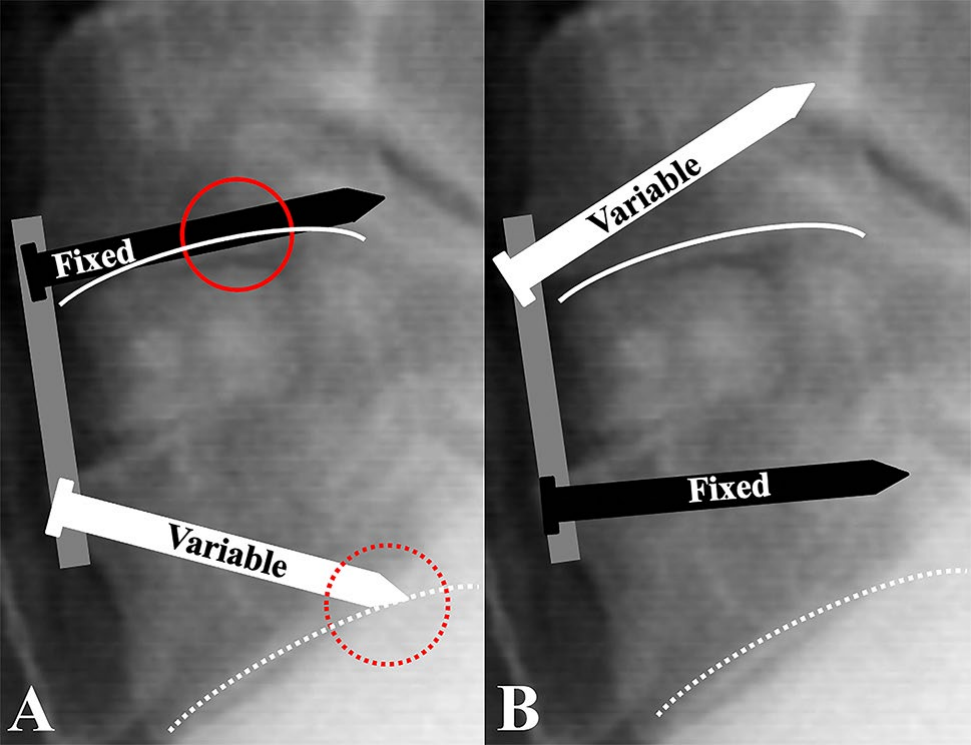
Avoiding endplate injury by selective caudal fixed screw construct. (**A**) Since the lower endplate of the cervical vertebra is shaped concavely in a sagittal plane fixed angled screw without high angulation has potential to injure the lower endplate of the cranial instrumented vertebra (red lined circle). Furthermore, due to the lordotic shape of cervical spine, variable screws are often inserted with more angulation than intended which could lead to injury of the lower endplate of the caudal instrumented vertebra (red dotted circle). (**B**) By using variable screws at cranial vertebra with greater angulation and fixed screw at caudal vertebra with less angulation, the risk of endplate injury could be avoided.

The ALOD more commonly occurs at the proximal adjacent segment, and a plate-adjacent distance of < 5 mm is known as a risk factor^9,10^. The ALOD limits motion at the adjacent segment and accelerates the degeneration of the level next to the adjacent level^11^. This justifies the need to increase the plate-disc space distance at the cranial level. In contrast, pseudarthrosis or implant failure most commonly occurs at the caudal level^6,17,18^. This can be explained by increased shear stress at the level near the cervicothoracic junction^25^. However, the ALOD risk is relatively low at the distal adjacent segment, which emphasizes the need for additional stability at the caudal level, whereas there is less need to increase plate-disc space distance^9,10^. Based on these previous findings, we attempted a hybrid construct using fixed screws selectively at the caudal level to increase its stability while inserting the variable screws at the cranial level. Another potential advantage of selective caudal screw construction is that endplate injury caused by screws can be avoided. Since the lower endplate of the vertebral body has concavity in the sagittal plane, inserting a fixed screw with less angulation has the potential to injure the lower endplate of the cranial vertebra. Furthermore, due to the lordotic shape of the cervical spine, screws are often inserted with greater angulation than intended, with a variable screw at the caudal level, which could injure the lower endplate of the caudal vertebra. Surgeons can decrease the risk of these endplate injuries by using variable screws at the cranial level and fixed screws at the caudal level (Fig. 4).

In the present study, ISM evaluated by dynamic radiography and bone bridging identified on CT was used to assess solid union. Riew et al. reported that the sensitivity and specificity for diagnosing pseudarthrosis were 87.1% and. 91.4%, respectively, with ISM^26^. Song et al. also demonstrated that bone bridging assessed on CT is a reliable marker of fusion^22^. Furthermore, subsidence was assessed by measuring the change of distance between the endplate of vertebral body and edge of the allograft in CT performed at 2 days and 1 year, postoperatively. Although subsidence is commonly assessed by the change in total interbody height or disc space height, this method would not be accurate when using allografts as interbody spacers because allografts themselves can change in height. Therefore, the change in the distance between the endplate of the vertebral body and the edge of the allograft was used for a more accurate assessment of subsidence.

The results of this study demonstrated that pseudarthrosis and subsidence most commonly occur at the caudal level. These results are consistent with the results of previous reports^6,17,18^. However, the fusion rates of selective fixed constructs and all variable constructs did not differ significantly. Although the locking mechanism at the screw-plate interface of the fixed screw was expected to increase the stability of the caudal segment, it did not lead to increased fusion rate. The ALOD and ASD rates were not significantly different between the SF and AV groups. The cranial and caudal plate-adjacent disc space distance and rate of patients with a plate-adjacent disc space distance of < 5 mm, which is a risk factor of ALOD, did not significantly differ between the two groups. Furthermore, clinical results, such as neck and arm pain VAS and NDI scores, did not significantly differ between the two groups.

Since the rates of pseudarthrosis, subsidence, ALOD, and ASD were similar in both groups, the selective fixed screw construct did not seem to provide additional advantage over the all variable screw constructs. The variable screws are more advantageous than the fixed screws at the point where the insertion angle is freely modifiable and a longer screw can be inserted with increased angle. In contrast, the advantage of the fixed screw is that it can be easily inserted with constant angulation. Based on the results of this study, screw types can be selected based on individual patient’s anatomy and surgeon’s experience, without concern for increased pseudarthrosis or subsidence caused by screw type. Oh et al. also reported that fusion rates of using the fixed and variable screws are similar. This is consistent with the results of this study^16^.

Further studies should be conducted to clarify the method to decrease the rate of pseudarthrosis at the caudal level, especially for multi-level surgery, since the results of the current study did not demonstrate significant results by screw construct difference. Although Lu et al. reported that pseudarthrosis at the caudal level can be decreased by selectively using low-dose bone morphogenic protein at the caudal level, there is still concern regarding complications caused by bone morphogenic protein for anterior cervical surgery^18^.

Previous studies have demonstrated that the number of fusion levels, bone graft type, plating, sex, age, smoking, greater preoperative segmental motion, and greater T1 sagittal slopes are related factors associated with pseudarthrosis after ACDF^27–30^. The result of the logistic regression analysis in this study also demonstrates that multi-level operation is a risk factor of pseudarthrosis, consistent with the results of previous studies. With increased fusion level, micromotion and contract stress also would have increased at the graft–bone interface, which could lead to pseudarthrosis^31^.

Our study has some limitations. First, this study had a limited sample size to assess the rate of ALOD or ASD. Second, there was a temporal difference in the type of operation performed. Although all operations were performed by a single surgeon at a single institute, unidentifiable factors due to time difference could have affected the results. Third, insertion angles and lengths of the screw were not considered as factors. However, a previous study demonstrated that screw insertion angle does not affect subsidence or fusion rate^16^. Finally, this study has a potential bias due to the retrospective nature of this study.

In conclusion, the fusion rates, subsidence, patient-reported outcome measurements, plate-adjacent disc space distance, ALOD, and ASD were not significantly different between the selective caudal fixed screw and all variable screw constructs. The stability provided by the locking mechanism of the fixed screw did not lead to an increased fusion rate. Therefore, it would be better to select screws based on individual patient’s anatomy and surgeon’s experience without concern for increased complications caused by screw type. This study demonstrates provisional results of comparison between all variable screw constructs and selective caudal fixed screw constructs in ACDF. Further clarification with a larger sample size is warranted.

## Data Availability

The datasets generated during and/or analyzed during the current study are available from the corresponding author on reasonable request.
